# Notes and News

**Published:** 1895-05-11

**Authors:** 


### Notes and News.

(Collected and Communicated.)

Cases of the plague have again occurred at Hong Kong.

At the conversazione of the Medical Society on Monday, 20th inst., Mr. Pearce Gould will deliver the oration on "The Recent Evolution of Surgery."

We regret that by a printer's error the date of the festival dinner of the Royal Medical Benevolent College was last week announced for the 5th instead of the 15th of May. Moreover, since the announcement the place of dining has been altered. The dinner will take place on May 15th, at the Imperial Institute.

On the 24th ult. the Rev. John Henn, of Manchester, was presented with a piece of silver plate and a sum of money—the total subscriptions amounted to £180—in recognition of his services to the Manchester Hospital Sunday Fund. Mr. Henn did good service in Manchester during twenty-five years in connection with this fund, and has well earned the testimonial in question. We are sorry to see, however, that in his reply he allowed himself to state: "Different people laid claim to the honour of originating the movement, but until the effort made in Manchester it was pretty certain that 'Hospital Sunday' was an institution unknown to the country." In The Hospital for October 13th, 1894, page 33, it is conclusively proved that, as Mr. Henn well knows, Hospital Sunday was known and in full progress at Birmingham in 1865, or five years before any movement of the kind was commenced in Manchester at all. Mr. Henn did really excellent work as honorary secretary to the Manchester Fund, and it is matter for regret that he should have permitted himself to utter the erroneous statement referred to upon an occasion when his fellow townsmen were testifying to their appreciation of the work he has done so well at Manchester.

Baron de Hirsch has sent a donation of £300 to King's College Hospital.

It is proposed to institute a memorial to Dr. Hack Tuke to take the form of a prize or medal for the encouragement of the study of psychological medicine.

The fourteenth annual meeting of the Mission to Deep Sea Fishermen will be held in Exeter Hall on Tuesday, May 14th, at three p.m.

Mr. John Whitaker Hulke, late president of the Royal College of Surgeons, has left property to the Middlesex Hospital for the establishment of a convalescent branch to be called after his name.

The Metropolitan Asylums Board, and the Local Government Board, are investigating the charge made that patients are discharged too soon from the fever hospitals.

We are informed that the Princess of Wales has consented to open a grand bazaar in aid of St. Mary's Hospital, Paddington, W., at the Portman Rooms, on Thursday, June 27th next.

The Therapeutic Committee of the British Medical Association have drawn up and are circulating a list of drugs of doubtful value, asking members to fill it up with a view to the revision of the British Pharmacopœia.

The proceeds of the bazaar held in aid of the Derbyshire Royal Infirmary have reached the sum of £4,300. The marked success of bazaars in the country, as compared with that which attends the same functions in the metropolis, suggest that other means more attractive should be devised for securing funds for London charities.

Her Royal Highness the Princess Mary Adelaide, Duchess of Teck, has graciously consented to visit the Chelsea Hospital for Women, on Wednesday, May 22nd, at 4 p.m., when Her Royal Highness will open and name the "Mary Adelaide" Ward, also receive purses of money from ladies who are willing to aid this institution. Purses may be obtained from the secretary at the hospital.

In the small medical school at Hyderabad many nationalities are represented by the students of both sexes. The women share equal advantages with the men; and as the hospital is chiefly used for surgical cases, considerable experience in operations is gained, as well as in the administration of anæsthetics. Dr. Laurie and Dr. Patrick Helier number amongst their pupils Parsees, Mahommedans, Hindus, Eurasians, and English.

The annual meeting of the British Medical Association will bring a number of distinguished foreign visitors to London in response to invitations already accepted for July 30th and following days. Sir Russell Reynolds, as President of the Association, will deliver the opening address at the Imperial Institute, and this will be followed by a conversazione. Exeter Hall will be the scene of the general meetings, whilst King's College and the Examination Hall of the Royal College of Physicians and Surgeons will be used for the majority of the sections.

